# *Microascaceae* from the Marine Environment, with Descriptions of Six New Species

**DOI:** 10.3390/jof10010045

**Published:** 2024-01-05

**Authors:** Meng-Meng Wang, Shi-Yu Yang, Qi Li, Yao Zheng, He-He Ma, Ye-Hui Tu, Wei Li, Lei Cai

**Affiliations:** 1 College of Science, Shantou University, Shantou 515063, China; mmwang@stu.edu.cn (M.-M.W.); shiyuyang_stu@126.com (S.-Y.Y.); cheryli@stu.edu.cn (Q.L.); 22yhtu1@stu.edu.cn (Y.-H.T.); 2 Guangdong Provincial Key Laboratory of Marine Biotechnology, Shantou University, Shantou 515063, China; 3 Guangdong Provincial Key Laboratory of Marine Disaster Prediction and Prevention, Shantou University, Shantou 515063, China; 4 College of Marine Life Sciences, Ocean University of China, Qingdao 266005, Chinamaheouc@163.com (H.-H.M.); 5 State Key Laboratory of Mycology, Institute of Microbiology, Chinese Academy of Sciences, Beijing 100101, China

**Keywords:** marine fungi, new species, *Microascaceae*, species diversity

## Abstract

Most reported members of *Microascaceae* that have been reported originate from the terrestrial environment, where they act as saprobes or plant pathogens. However, our understanding of their species diversity and distribution in the marine environment remains vastly limited, with only 22 species in nine genera having been reported so far. A survey of the fungal diversity in intertidal areas of China’s mainland has revealed the discovery of several *Microascaceae* strains from 14 marine algae and 15 sediment samples. Based on morphological characteristics and LSU-ITS-*tef1*-*tub2* multilocus phylogeny using Bayesian inference and maximum likelihood methods, 48 strains were identified as 18 species belonging to six genera. Among these, six new species were discovered: *Gamsia sedimenticola*, *Microascus algicola*, *M. gennadii*, *Scedosporium ellipsosporium*, *S. shenzhenensis,* and *S. sphaerospermum*. Additionally, the worldwide distribution of the species within this family across various marine habitats was briefly reviewed and discussed. Our study expands the knowledge of species diversity and distribution of *Microascaceae* in the marine environment.

## 1. Introduction

The family *Microascaceae* (*Microascales*, *Sordariomycetes*, and *Ascomycota*) was originally erected in 1951 by Luttrell to accommodate the genus *Microascus*, characterised as having beaked ascocarps with evanescent asci disposed irregularly throughout the centrum [1]. Later in 1970, Malloch reviewed known species, introduced two new species, and then illustrated the new concepts of this family, including ascocarps, ascospores, and complex hyaline to brightly coloured, conidiophores that are phialide bearing [2]. Over the past several decades, advances in morphology and multilocus phylogenetic analyses have led to a better understanding of the taxonomy and species diversity of this family. Species in this family are mainly characterised by their annellidic asexual morphs with dry aseptate conidia, as well as their sexual morphs that form cleistothecial or perithecial, carbonaceous ascomata producing reniform, lunate, or triangular ascospores with or without germ pores [3]. Currently, about 290 species in 23 genera are accepted in *Microascaceae* [4]. The majority of these species are found in terrestrial environments and act as saprobes or pathogens of plants, and also as opportunistic pathogens of humans with some exhibiting intrinsic resistance to antifungal agents [5].

However, there is limited information available on the species diversity and distribution of *Microascaceae* in the marine environment. In 1973, a new species, *Scopulariopsis halophilica* Tubaki, was introduced and described from the macroalgae *Undaria pinnatifida* collected from Japan [6]. This is, to our knowledge, the first microascaceous fungus to be isolated from the marine environment. Since then, several studies using culture-dependent approaches have documented the wide distribution of microascaceous fungi from intertidal zones of European and tropical regions, and marginal seas of China, with a total of 22 species reported in nine genera (*Acaulium*, *Cephalotrichum*, *Microascus*, *Petriella*, *Pseudallescheria*, *Pseudoscopulariopsis*, *Scedosporium*, *Scopulariopsis,* and *Wardomyces*) [7,8,9,10]. Several culture-independent approaches using ITS1 or ITS2 rDNA metabarcoding have uncovered substantial occurrences of this family in the seas surrounding China [11,12,13]. This is fortified by the identification of 109 microascaceous OTUs (operational taxonomic units), which were annotated into 13 genera, and extracted from intertidal sediments from China’s mainland [14]. Five microascaceous OTUs were also detected from the seawater samples collected from the Western Pacific Ocean [15]. These findings indicate the potential global distribution of microascaceous fungi within the ocean.

Marine fungi are capable of producing a variety of secondary metabolites such as those with antimicrobial properties [16]. The *Microascaceae* fungi recovered from the marine environment have demonstrated great potential in industrial and agricultural applications. A new monoterpenoid compound, Scopuquinolone B, was first isolated from a coral-derived *Scopulariospis* sp. isolate LF580 [17]. Proteomic analysis of *Sc. brevicaulis* from marine sponges identified thousands of proteins and diverse biosynthetic enzyme complexes [18]. Several species, e.g., *Micoascus trigonosporus*, displayed a highly lethal effect on agricultural pests and a significant inhibitory effect on pathogens of plant diseases [19]. It is necessary to obtain more *Microascaceae* fungi from the marine environment and to promote more research about the physiology, biochemistry, metabolites, and ecological functions of this group of fungi.

Most of the known culturable fungal species have been found on various substrates collected from the intertidal region, such as seagrasses, seaweeds, mangrove plants, sediments, and driftwood [7,8,9,10], indicating that the intertidal region provides an ideal shelter to host numerous and diverse fungi. In a survey of fungal diversity in the intertidal areas of China’s mainland, 48 microascaceous strains were isolated from 14 marine algae and 15 sediment samples collected from 22 locations. Our aims for this study are (1) to identify species and clarify the phylogenetic relationships of these fungi, (2) to identify and describe new species of *Microascaceae*, and (3) to review species diversity, host/habitat, and geographic distribution of *Microascaceae* in the marine environment.

## 2. Materials and Methods

### 2.1. Sample Collection and Fungal Isolation

**Figure 1:**
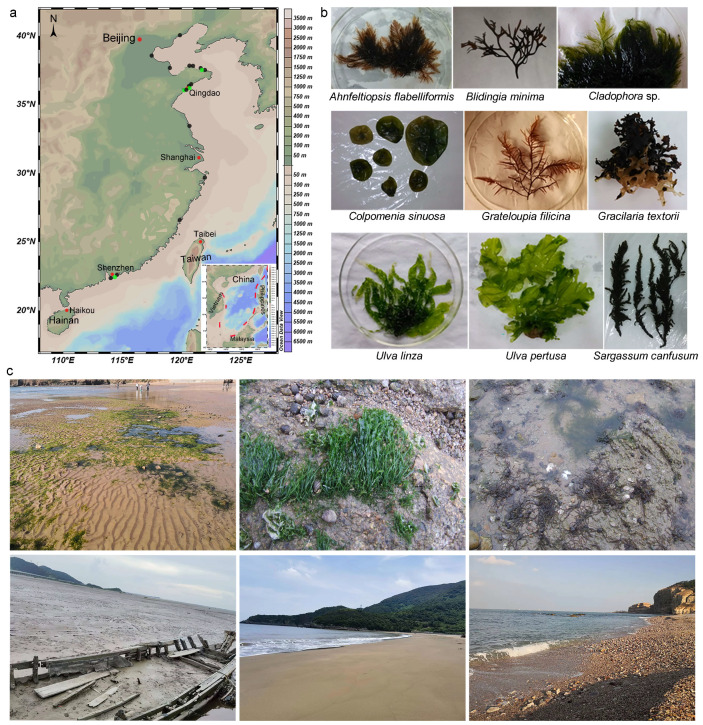
Locations of the 14 marine algae (green dot) and 15 sediments (black dot) samples from the intertidal areas of mainland China (**a**), the thallus of algal hosts of the microascaceous fungi (**b**), and the typical algal habitats and the typical mudflat, sand beach, and gravel beach in the intertidal zones (**c**).

Algae and sediment samples were collected from intertidal zones of Fujian, Guangdong, Jiangsu, Liaoning, Shandong, and Zhejiang provinces, and Tianjin city (Figure 1). For algae, six asymptomatic thalli of each algae species from each site were collected and put into sterilised plastic bottles. Species identification of algae was carried out following morphological descriptions by Zeng [20]. For sediment samples, a 200 g sediment sample was collected from each site and placed into a sterilised sampling bag. The samples were placed into a low-temperature storage box with gel packs and brought back to the laboratory as soon as possible.

Fungi were isolated from algae using the dilution-plate method [21] and tissue-isolation method, with some modifications. Each algae sample was washed with sterilised seawater, cut into segments of about 0.5 × (0.2–0.5) cm, and surface sterilised by dipping in 70% ethanol for 5 s followed by immersion in 4% NaOCl for 60 s and washed with sterile distilled water for 10 s [22]. Using the dilution-plate method, approximately 1 g of the segments were placed in a sterilised mortar and ground to tissue homogenate with 10 mL of sterilised seawater. The suspension was moved into a sterilised tube, diluted to a series of concentrations (10^−1^, 10^−2^, 10^−3^, and 10^−4^), and, finally, spread onto isolation media with three replicates. Using the tissue-isolation method, after cutting into segments and surface sterilization, 6–10 algal segments were spread on the isolation media with three replicates. Five types of isolation media, namely Martin medium (MM; each 1 L medium containing peptone 5.0 g, dextrose 10.0 g, monopotassium phosphate 1.0 g, magnesium sulfate 0.5 g, chloramphenicol 0.1 g, rose Bengal 0.033 g, agar 20.0 g, and seawater 1 L), 1/10 potato dextrose agar (1/10 PDA; each 1 L medium containing potato 20.0 g, dextrose 2.0 g, agar 20.0 g, and seawater 1 L), 1/5 malt extract agar (1/5 MEA; each 1 L medium containing malt extract 4.0 g, peptone 0.2 g, dextrose 4.0 g, agar 20.0 g, and seawater 1 L), corn meal agar (CMA; each 1 L medium containing corn meal 30.0 g, agar 20.0 g, and seawater 1 L), and yeast extract peptone glucose agar (YPG; each 1 L medium containing yeast extract 1.25 g, peptone 1.25 g, dextrose 4.0 g, agar 20.0 g, and seawater 1 L) containing ampicillin (500 mg/L) and streptomycin (500 mg/L), were selected and used for fungal isolation in this study.

Fungi were isolated from sediments using the dilution-plate method [21] and the direct-isolation method, with some modifications. In the dilution-plate method, 10 g of sediment from each sample were suspended in 90 mL of sterile seawater in a 100 mL triangular glass flask and cultivated by shaking at 150 rpm/min at 25 °C for 20 min. The suspension was diluted to a series of concentrations (10^−1^, 10^−2^, 10^−3^, and 10^−4^) and plated on five types of isolation media with three replicates. Using the direct-isolation method, approximately 1 g of each sediment sample was spread directly on five types of isolation media with three replicates.

All plates were incubated at room temperature and examined every 2 days using an Olympus SZX7 stereomicroscope for fungal hyphae. Individual colonies were picked up with a sterilised needle and transferred onto fresh PDA plates on the benchtop. All the cultures were incubated at room temperature after 7 days and then were purified using an optimised protocol for single-spore isolation [23]. All isolates examined in this study were deposited in Wei Li’s personal culture collection (WL). Type specimens of new species were deposited in the Fungarium of the Institute of Microbiology (HMAS), with the ex-type living cultures in the China General Microbiological Culture Collection Center (CGMCC).

### 2.2. Morphological Observation

The isolates studied were incubated on synthetic nutrient-poor agar plates (SNA) [24], PDA, and oatmeal agar (OA). After 7 days of incubation in the dark, culture characteristics, including colony morphology, pigmentation, and odour were observed. Colours were assessed according to the colour charts of Kornerup and Wanscher (1978) [25]. Micromorphological characteristics were examined and photo-documented using water as a mounting medium under an Olympus BX53 microscope with differential interference contrast (DIC) optics. For each species, respectively, 30 conidiophores, 30 conidiogenous cells, 30 chlamydospores, and 50 microconidia were mounted and measured randomly.

### 2.3. DNA Extraction and Amplification

Genomic DNA was extracted from fungal mycelia grown on PDA, using a modified CTAB protocol as described in Guo et al. (2000) [26]. Four loci, including partial large-subunit ribosomal RNA (LSU), 5.8S nuclear ribosomal RNA gene with the two flanking internal transcribed spacer (ITS) regions, partial translation elongation factor (*tef1*), and partial β-tubulin (*tub2*), were amplified using primer pairs LR0R/LR5 [27], ITS5/ITS4 [28], EF1-983F/EF1-2218R [29], and Bt2a/Bt2b [30], respectively. Amplification reactions were performed in a reaction volume containing 12.5 μL of 2 × Taq PCR Master Mix (Vazyme Biotech Co., Ltd., Nanjing, China), 1 μL each of 10 μM primers, and 1 μL of the undiluted genomic DNA, adjusted to a final volume of 25 μL with distilled deionised water (Dongsheng, EDC810, China). PCR parameters were as follows: 94 °C for 10 min, followed by 35 cycles of 94 °C for 30 s, 50 (for LSU)/54 (for ITS)/57 (for *tef1* and *tub2*) °C for 30 s, 72 °C for 30 s, and a final elongation step at 72 °C for 10 min. The PCR products were visualised on 1% agarose electrophoresis gel. Sequencing was performed bidirectionally and conducted by the BGI Write Company (Beijing, China). Consensus sequences were obtained using SeqMan of the Lasergene software package v. 14.1 (DNAstar, Madison, WI, USA).

### 2.4. Phylogenetic Analyses

The sequences of the *Microascaceae* strains examined in this study and the reference strains are listed in Table 1. For each locus, sequences were aligned using MAFFT v. 7 [31], and the alignments were manually adjusted where necessary. The best-fitting nucleotide-substitution models according to the Akaike Information Criterion (AIC) were selected using jModelTest v. 2.1.7 [32,33]. Alignments derived from this study were deposited in TreeBASE (submission ID 30581), and taxonomic novelties were deposited in FungalNames.

Phylogenetic analyses of the combined dataset were performed using Bayesian inference (BI) and maximum-likelihood (ML) methods. The BI analyses were conducted using MrBayes v. 3.2.1 [34] following the protocol of Wang et al. (2019) [35], with optimisation of each locus treated as a partition in combined analyses, based on the Markov Chain Monte Carlo (MCMC) approach [36]. All characters were equally weighted, and gaps were treated as missing data. The stationarity of the analyses was determined by examining the standard deviation of split frequencies (<0.01) and –ln likelihood plots in AWTY [37]. The ML analyses were conducted using PhyML v. 3.0 [38], with 1000 bootstrap replicates. The general time reversible model was applied with an invariable gamma-distributed rate variation (GTR+I+G).

## 3. Results

### 3.1. Phylogenetic Analyses

**Figure 2:**
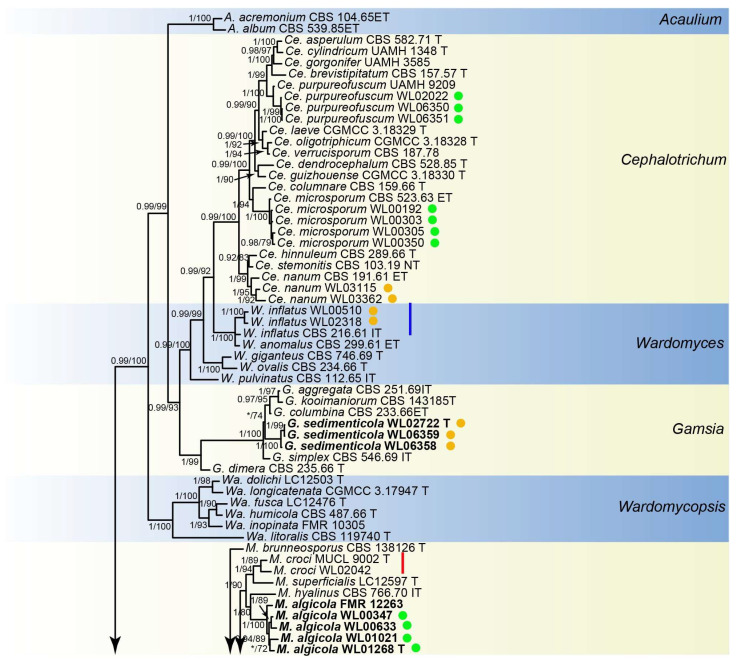

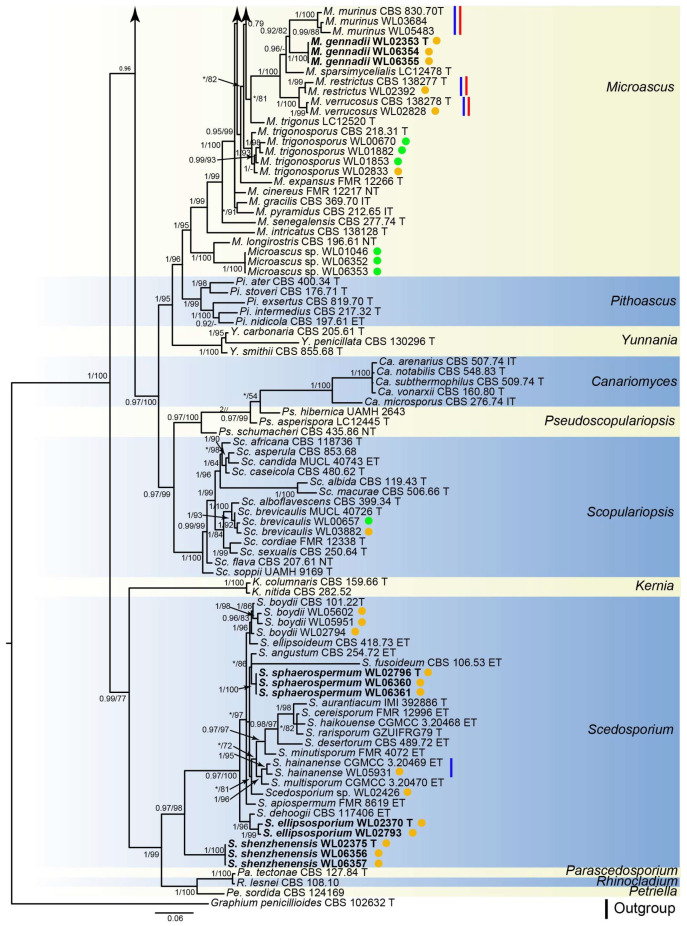
Fifty percent majority rule consensus tree from a Bayesian analysis based on a four-locus combined dataset (LSU-ITS-*tef1*-*tub2*) showing the phylogenetic relationships of genera within the family *Microascaceae*. The Bayesian posterior probabilities (PP > 0.9) and PhyML bootstrap support values (BS > 50%) are displayed at the nodes (PP/BS). The tree was rooted to *Graphium penicillioides* (CBS 102632 T). Ex-type cultures are indicated with “T”, epi-type with “ET”, iso-type with “IT”, and neo-type with “NT”. New species introduced in this paper are marked in bold. New records in the marine environment are marked in the blue line and new records in China in the red line. Strains newly isolated in this study are marked in green dots (isolates from algae) and brown dots (isolates from sediment).

Analyses of the *Microascaceae* phylogeny were conducted by using a combined LSU (897 bp), ITS (665 bp), *tef1* (924 bp) and *tub2* (539 bp) dataset. For the BI analysis, the GTR+I+G model was selected for the LSU, ITS, *tef1* and *tub2* loci. The phylogeny showed that our isolates were clustered into 18 species in six genera of Microascaceae, namely *Cephalotrichum* (3 species), *Gamsia* (1), *Microascus* (7), *Scedosporium* (5), *Scopulariopsis* (1), and *Wardomyces* (1), including six new species (Figure 2).

### 3.2. Species List and Taxonomy

***Cephalotrichum*** Link, Mag. Gesell. naturf. Freunde, Berlin 3(1-2): 20 (1809).

***Cephalotrichum microsporum*** (Sacc.) P.M. Kirk, in Kirk and Spooner, Kew Bull. 38(4): 578 (1984).

Examined isolates: CHINA, Shandong Province, Qingdao city, from an unidentified red alga collected from a sand beach, April 2010, Y.T. Peng and K.M. Sun (WL00192); Weihai city, from unidentified green algae, June 2010, K.M. Sun and W. Li (WL00303, WL00305); Weihai city, from an unidentified brown alga, June 2010, K.M. Sun and W. Li (WL00350).

***Cephalotrichum nanum*** (Ehrenb.) S. Hughes, Can. J. Bot. 36: 744 (1958).

Examined isolates: CHINA, Zhejiang Province, Ningbo city (121°46′36.72″ E, 29°58′15.42″ N), from intertidal sediment of a mudflat, July 2014, X.M. Bian and W. Li (WL03115, WL03362).

***Cephalotrichum purpureofuscum*** (S. Hughes) S. Hughes, Can. J. Bot. 36: 744 (1958).

Examined isolates: CHINA, Guangdong Province, Shenzhen city, from *Sargassum canfusum* collected from a gravels beach, May 2014, M.M. Wang and W. Li (WL02022, WL06350, WL06351).

***Gamsia*** M. Morelet, Ann. Soc. Sci. Nat. Arch. Toulon et du Var 21: 105 (1969).

**Figure 3:**
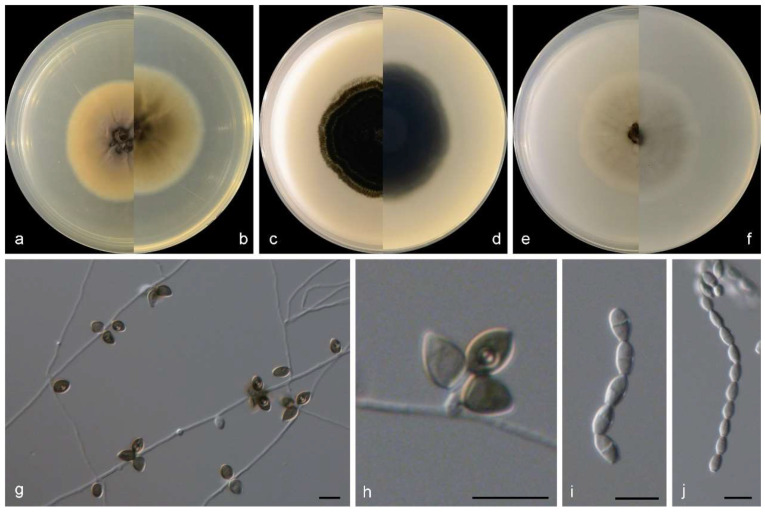
Morphological characters of *Gamsia sedimenticola* (from ex-type WL02722). (**a**–**f**): colonies on PDA, OA and SNA after 7d; (**g**,**h**): Conidiogenous cells; (**i**,**j**): Conidia in chains. Bars: (**g**–**j**) = 10 μm.

***Gamsia sedimenticola***M.M. Wang, W. Li and L. Cai sp. nov., Figure 3.

FungalNames: FN 571622.

Etymology: named after the habitat of the type specimen, sediment.

Typus: CHINA, Shandong Province, Qingdao city (120°19′09.12″ E, 36°03′38.64″ N), from intertidal sediment of a mudflat, June 2014, X.M. Bian and W. Li (HMAS352501, holotype designated here, dried culture on SNA; culture ex-type CGMCC3.25342 = WL02722).

Sexual morph not observed. Asexual morph on PDA and OA conidiophores reduced to conidiogenous cells, polyblastic, subcylindrical to cylindrical with a swollen apical part, 2–2.5 × 2–3.5 μm, and hyaline to somewhat darkening at the apex, smooth- and thin-walled, with 1–3 apical conidiogenous loci. Conidia 0–1-septate, ovoid to broadly ellipsoidal, with a rounded-to-pointed apex, flat at the base, pale to dark brown, 7–9 × 5–6.5 μm, smooth and thick walled, and often with a conspicuous longitudinal germ slit.

Culture characteristics—colonies on PDA are 30–40 mm diam in 7 d at 25 °C, flat, felty to floccose, and olive green near the centre, with a white regular margin; reverse pale olive green near the centre and white near the margin. Colonies on OA are 30–40 mm diam in 7 d at 25 °C, margin regular, flat, and dark olive green; the reverse is dark olive green. Colonies on SNA are 30–35 mm diam in 7 d at 25 °C, flat, with scarce aerial mycelia, and pale olive green near the centre, with a white regular margin; reverse pale olive green near the centre, white near the margin.

Other examined isolates: CHINA, Shandong Province, Qingdao city (120°19′09.12″ E, 36°03′38.64″ N), from intertidal sediment of a mudflat, June 2014, X.M. Bian and W. Li (WL06358); ibid. (WL06359).

Notes: The genus *Gamsia* was erected to accommodate the *Wardomyces* species that form 1-septate annelloconidia [39]. Sandoval-Denis et al. demonstrated that the lack of well-differentiated conidiophores and the conidial arrangement with large apical clusters justifies the separation of *Gamsia* from *Wardomyces* [40]. Five species are currently accepted in *Gamsia* [39,40,41,42,43], and one new species, *G. sedimenticola,* was added in this study. Phylogenetically, *G. sedimenticola* is closely related to *G. aggregate* (Malloch) Kiffer and M. Morelet, *G. columbina* (Demelius) Sand.-Den., Guarro and Genéand *G. kooimaniorum* Sand.-Den. (Figure 2), but differs from *G. aggregate* and *G. kooimaniorum* by 29 and 24 bp in the LSU-ITS sequences, and from *G. columbina* by 34 bp in the LSU-ITS-*tef1*-*tub2* dataset, respectively. Morphologically, the four species exhibit marked differences in characters of aerial conidiophores (reduced to conidiogenous cells, polyblastic, subcylindrical to cylindrical with a swollen apical part, 2–2.5 × 2–3.5 μm in *G. sedimenticola* vs. borne singly or in small clusters on short branches, flask-shaped, 10–35 × 3–4 μm in *G. aggregate*, usually unbranched, 0−1-septate, 3–15 × 1.5–3 μm in *G. columbina* and unbranched or rarely laterally branched once, produced abundantly borne laterally and singly on the aerial hyphae, 0−1(−2)-septate, 12–28 × 1.5–7 μm in *G. kooimaniorum*), and the shape, size, and colour of conidia (7–9 × 5–6.5 μm, ovoid to broadly ellipsoidal, pale to dark brown in *G. sedimenticola* vs. 8–10.5 × 3.5–5 μm, ellipsoidal, rounded or apiculate/hyaline in *G. aggregate*, 5–10.5 × 2.5–5.5 μm, oval, smooth/hyaline in *G. columbina* and 5.5–10.5 × 4–7 μm, ovoid to broadly ellipsoidal, and pale to dark brown in *G. kooimaniorum*) [40,41,42,43]. In addition, *G. sedimenticola* was isolated from intertidal sediment in China, differing from *G. aggregate* from the dung of a carnivore in the USA, *G. columbina* from air, soil, decaying wood, and milled rice in Austria, Germany, Japan, and the Netherlands, and *G. kooimaniorum* from the soil in Netherlands [40,41,42,43].

***Microascus***Zukal, Verh. Kaiserl.-Königl. zool.-bot. Ges. Wien 35: 342 (1886).

**Figure 4:**
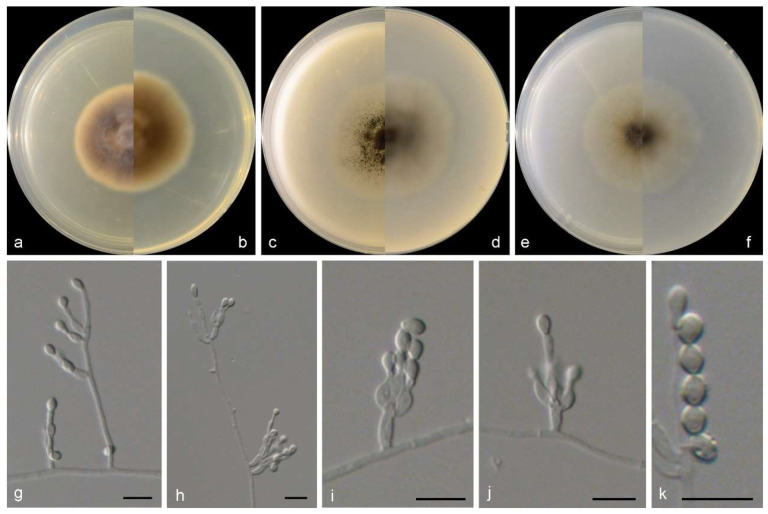
Morphological characters of *Microascus algicola* (from ex-type WL01268). (**a**–**f**): colonies on PDA, OA and SNA after 7 d; (**g**–**j**): Conidiophores and conidiogenous cells; (**k**): Conidia in chains. Bars: (**g**–**k**) = 10 μm. Bars: (**g**–**k**) = 10 μm.

***Microascus algicola***M.M. Wang, W. Li and L. Cai sp. nov., Figure 4.

FungalNames: FN 571623.

Etymology: named after the host of type specimen, algae.

Typus: CHINA, Shandong Province, Qingdao city, from *Grateloupia filicina* collected from a gravels beach, unknown date, M.M. Wang and W. Li (HMAS352502, holotype designated here, dried culture on SNA; culture ex-type CGMCC3.25343 = WL01268).

Sexual morph not observed. Asexual morphs on PDA, OA, and SNA conidiophores are simple, straight, septate, branched or rarely unbranched, and hyaline. Conidiogenous cells in whorls of 2–3 on the apex of conidiophores, or rarely solitary on aerial hyphae, lageniform to ampulliform, straight or slightly curved, pale brown, 5–11 × 2.5–4 µm. Conidia in long chains, ellipsoidal, smooth to slightly rough, thick walled, hyaline to pale brown, and 3.5–6 × 3–5 µm.

Culture characteristics—colonies on PDA are 35–45 mm diam in 7 d at 25 °C, flat, felty to floccose, and greyish brown near the centre, with a pale-brown regular margin; the reverse is greyish brown near the centre, white near the margin. Colonies on OA are 35–45 mm diam in 7 d at 25 °C, margin regular, flat, and dark olive green in the centre, with a white regular margin; the reverse is dark olive green near the centre and white near the margin. Colonies on SNA are 30–35 mm diam in 7 d at 25 °C, flat, with scarce aerial mycelia, and pale olive green near the centre, with a white regular margin; the reverse is pale olive green near the centre and with a white regular margin.

Other examined isolates: CHINA, Shandong Province, Weihai city, from an unidentified brown alga collected from a sand beach, June 2010, K.M. Sun and W. Li (WL00347); Qingdao city, from *Colpomenia sinuosa* from a sand beach, October 2011, Y.T. Peng, C.H. Feng, and C.L. Li (WL00633), from *Ahnfeltiopsis flabelliformis* from a sand beach, December 2014, M.M. Wang (WL01021).

Notes: phylogenetically, *Microascus algicola* is most closely related to *M. hyalinus* (Malloch and Cain) Sand.-Den., Gené and Guarro (Figure 2), but differs by 233 bp in the four loci dataset. Morphologically, *M. algicola* could be distinguished in the shape and size of conidia (ellipsoidal, 3.5–6 × 3–5 µm), while *M. hyalinus* produces ovoid, 3.5–5 × 2–3.5 μm conidia [4]. In addition, currently, all known isolates of *M. algicola* was isolated from marine algae (listed above) in intertidal zones in China, while *M. hyalinus* was isolated from soil and dung in Europe and North America [44,45].

***Microascus croci*** (J.F.H. Beyma) Sand.-Den., Gené and Guarro, in Sandoval-Denis, Gené, Sutton, Cano-Lira, de Hoog, Decock and Guarro, Persoonia 36: 17 (2015).

Examined isolates: CHINA, Shandong Province, Qingdao city, from *Blidingia minima* from a gravels beach, May 2014, M.M. Wang and W. Li (WL02042).

**Figure 5:**
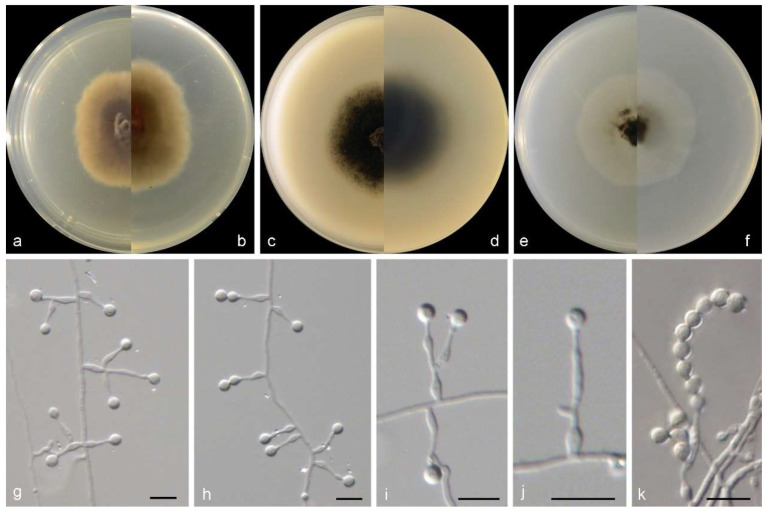
Morphological characters of *Microascus gennadii* (from ex-type WL02353). (**a**–**f**): colonies on PDA, OA, and SNA after 7 d; (**g**–**j**): Conidiophores and conidiogenous cells; (**k**): Conidia in chains. Bars: (**g**–**k**) = 10 μm.

***Microascus gennadii*** M.M. Wang, W. Li, and L. Cai sp. nov., Figure 5.

Fungal names: FN 571624.

Etymology: named after the ellipsoidal conidia of this species.

Typus: CHINA, unknown location, from intertidal sediment, May 2014, X.M. Bian and W. Li (HMAS352503, holotype designated here, dried culture on SNA; culture ex-type CGMCC3.25344 = WL02353).

Sexual morph is not observed. Asexual morph on PDA and OA conidiophores are simple, straight, septate, branched or rarely unbranched, and hyaline. Conidiogenous cells are solitary on aerial hyphae or in whorls of 2–3 on the apex of conidiophores, lageniform to ampulliform, straight or slightly curved, pale brown, and 9–14 × 2–3.5 µm. Conidia are in long chains, ellipsoidal, smooth to slightly rough, thick-walled, hyaline, and 4–8 × 3–6 µm.

Culture characteristics—colonies on PDA are 35–45 mm diam in 7 d at 25 °C, flat, felty to floccose, and pale grey near the centre, with a pale brown irregular margin; the reverse is pale brown near the centre and white near the margin. Colonies on OA are 35–40 mm diam in 7 d at 25 °C, margin regular, flat, and dark olive green, with a white regular margin; the reverse is dark olive green near the centre and white near the margin. Colonies on SNA are 30–40 mm diam in 7 d at 25 °C, flat, with scarce aerial mycelia, and pale olive green near the centre, with a white regular margin; the reverse is pale olive green near the centre, with a white regular margin.

Other examined isolates: CHINA, unknown location, from intertidal sediment, May 2014, X.M. Bian and W. Li (WL06354); ibid. (WL06355).

Notes: phylogenetically, *Microascus gennadii* is closely related to *M. murinus* (Samson and Klopotek) Sand.-Den., Gené and Guarro (Figure 2), but differs by 241 bp in the four loci dataset. Morphologically, the two species are distinct in the shape and size of conidia (ellipsoidal, 4–8 × 3–6 µm in *M. gennadii* vs. cylindrical, 4–6 × 1.5–2 μm in *M. murinus*) [5].

***Microascus croci*** (Samson and Klopotek) Sand.-Den., Gené and Guarro, in Sandoval-Denis, Gené, Sutton, Cano-Lira, de Hoog, Decock and Guarro, Persoonia 36: 21 (2015).

Examined isolates: CHINA, Shandong Province, Yantai city (120°42′07.50″ E, 37°48′41.22″ N), from the intertidal sediment of a gravel beach, June 2014, X.M. Bian and W. Li (WL03684); Qingdao city (120°11′42.05″ E, 36°3′45.58″ N), from the intertidal sediment of a gravel beach, August 2014, Y. Zheng, H.P. Yang and W. Li (WL05483).

***Microascus restrictus*** Sand.-Den., Gené and Deanna A. Sutton, in Sandoval-Denis, Gené, Sutton, Cano-Lira, de Hoog, Decock and Guarro, Persoonia 36: 21 (2015).

Examined isolates: CHINA, Liaoning Province, Huludao city (120°47′33.34″ E, 40°36′14.56″ N), from the intertidal sediment of a sand beach, May 2014, X.M. Bian and W. Li (WL02392).

***Microascus trigonosporus*** C.W. Emmons and B.O. Dodge, Mycologia 23(5): 317 (1931).

Examined isolates: CHINA, Shandong Province, Qingdao city, from *Cladophora* sp. from a sand beach, October 2010, Y.T. Peng, C.H. Feng and C.L. Li (WL00670); ibid., from *Ulva linza* from a sand beach, April 2014, M.M. Wang (WL01853); ibid., from *Gracilaria textorii* from sand beach, June 2014, M.M. Wang (WL01882); ibid., from the intertidal sediment of a sand beach, X.M. Bian and W. Li (WL02833).

***Microascus verrucosus*** Sand.-Den., Gené and Cano, in Sandoval-Denis, Gené, Sutton, Cano-Lira, de Hoog, Decock and Guarro, Persoonia 36: 23 (2015).

Examined isolates: CHINA, Shandong Province, Dongying city (119°09′45.18″ E, 37°45′34.86″ N), from the intertidal sediment of a mudflat, January 2014, M.M. Wang (WL02828).

***Scedosporium*** Sacc. ex Castell. and Chalm., Manual of Tropical Medicine (London): 1122 (1919).

***Scedosporium boydii*** (Shear) Gilgado, Gené, Cano and Guarro, J. Clin. Microbiol. 46(2): 770 (2008).

Examined isolates: CHINA, Shandong Province, Qingdao city (120°19′06.25″ E, 36°03′34.13″ N), from the intertidal sediment of a sand beach, January 2014, X.M. Bian and W. Li (WL02794); Weihai city (122°10′13″E, 37°30′2″ N), from the intertidal sediment of a sand beach, November 2020, M.M. Wang and Y. Zheng (WL05602); Tianjin city (117°45′38″ E, 39°6′36.64″ N), from the intertidal sediment of a mudflat, April 2021, M.M. Wang and Y. Zheng (WL05951).

**Figure 6:**
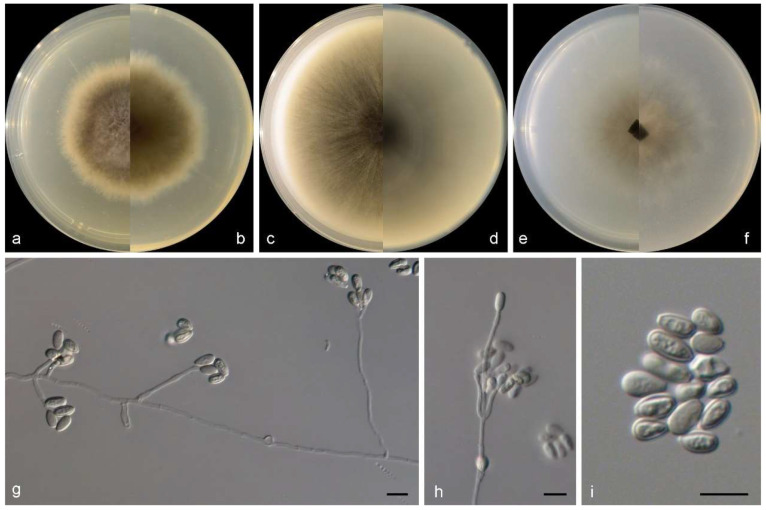
Morphological characters of *Scedosporium ellipsosporium* (from ex-type WL02370). (**a**–**f**): colonies on PDA, OA, and SNA after 7 d; (**g**,**h**): Conidiophores and conidiogenous cells; (**i**): Conidia. Bars: (**g**–**i**) = 10 μm.

***Scedosporium ellipsosporium*** M.M. Wang, W. Li and L. Cai sp. nov., Figure 6.

FungalNames: FN 571625.

Etymology: referring to the shape of conidia, ellipsoidal.

Typus: CHINA, Guangdong Province, Shenzhen city (114°20′45.63″ E, 22°36′01.03″ N), from intertidal sediment of a gravel beach, May 2014, X.M. Bian and W. Li (HMAS352504, holotype designated here, dried culture on SNA; culture ex-type CGMCC3.25345 = WL02370).

Sexual morph is not observed. Asexual morph on PDA and SNA conidiophores are solitary, often reduced to a single conidiogenous cell arising laterally from undifferentiated hypha, or stalked, bearing two or three conidiogenous cells at the top. Conidiogenous cells are annellidic, lateral or terminal, hyaline, smooth- and thin-walled, cylindrical to flask-shaped, and 10–40 × 1.5–2.5 µm, with several, distinct annellations at the top with age. Conidia aseptate, solitary, arranged in slimy masses, hyaline, ellipsoidal, flat at the base, and 5–9 × 3–5 µm.

Culture characteristics—colonies on PDA are 40–55 mm diam in 7 d at 25 °C, flat, felty to floccose, and olive green near the centre, with a white regular margin; the reverse is olive green near the centre, and white near the margin. Colonies on OA are 50–65 mm diam in 7 d at 25 °C, flat, and olive green near the centre, with a white regular margin; the reverse is olive green near the centre, and white near the margin. Colonies on SNA are 30–40 mm diam in 7 d at 25 °C, flat, with scarce aerial mycelia, and pale olive green near the centre, with a white regular margin; the reverse is pale olive green near the centre, with a white regular margin.

Other examined isolates: CHINA, Shandong Province, Qingdao city (120°19′09.12″ E, 36°03′38.64″ N), from intertidal sediment of a mudflat, June 2014, X.M. Bian and W. Li (WL02793).

Notes: phylogenetically, *Scedosporium ellipsosporium* is closely related to *S. dehoogii* Gilgado, Cano, Gené and Guarro (Figure 2), but differs by 42 bp in the ITS-*tub2* sequences. Morphologically, the two species could be distinguished in the colour, shape, and size of conidia (hyaline, ellipsoidal, and 5–9 × 3–5 µm in *S. ellipsosporium* vs. subhyaline or slightly grey, and usually ovate, 5–8 × 5–6 μm in *S. dehoogii*) [46].

***Scedosporium hainanense*** Zhi Y. Zhang, Y.F. Han and Z.Q. Liang, in Zhang, Shao, Li, Chen, Liang, Han, Huang and Liang, Microbiology Spectrum 9(2): e00867-21, 16 (2021).

Examined isolates: CHINA, Jiangsu Province, Yancheng city (119°52′32″ E, 34°27′27″ N), from intertidal sediment of a mudflat, April 2021, Z.Q. Zeng and C. Liu (WL05931).

**Figure 7:**
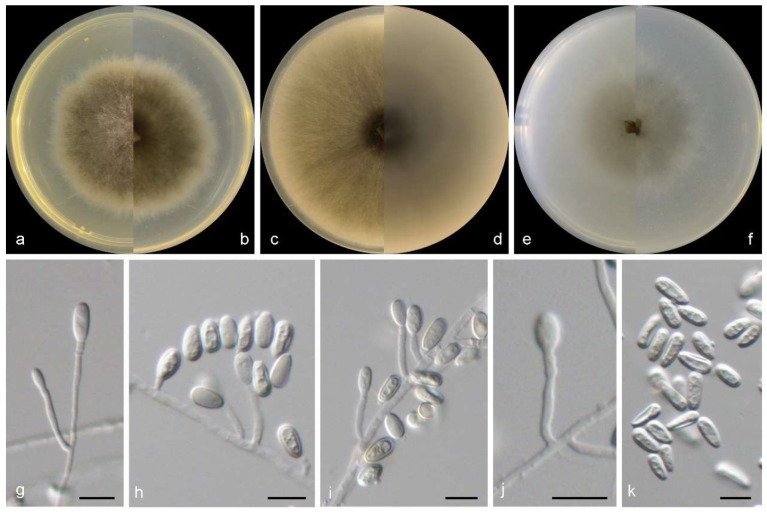
Morphological characters of *Scedosporium shenzhenensis* (from ex-type WL02375). (**a**–**f**): colonies on PDA, OA, and SNA after 7 d; (**g**–**j**): Conidiophores and conidiogenous cells; (**k**): Conidia. Bars: (**g**–**k**) = 10 μm.

Notes: Phylogenetically, *Scedosporium shenzhenensis* formed a distinct basal clade in *Scedosporium* (Figure 2). Considering the typical *Scedosporium* morphology of our isolates (Figure 8), we tentatively placed this species in the genus *Scedosporium*. Morphologically, *S. shenzhenensis* is similar to *S. aurantiacum* Gilgado, Cano, Gené and Guarro, *S. dehoogii*, *S. ellipsosporium* (Arx and Fassat.) McGinnis, A.A. Padhye and Ajello and *S. sphaerospermum* in the cylindrical to flask-shaped conidiogenous cells, but differs in the shape and size of conidia (ellipsoidal, 7–16 × 3.5–7 µm in *S. shenzhenensis* vs. obovoid, 6–10 × 3–5 µm in *S. aurantiacum*, obovoid, 5–8 × 5–6 µm in *S. dehoogii*, ellipsoidal, 5–9 × 3–5 µm in *S. ellipsosporium* and globose to ellipsoidal, 6–8 × 4–5 µm in *S. sphaerospermum*) [46,47,48].

***Scedosporium shenzhenensis*** M.M. Wang, W. Li and L. Cai sp. nov., Figure 7.

FungalNames: FN 571626.

Etymology: named after the location of the type specimen, Shenzhen.

Typus: CHINA, Guangdong Province, Shenzhen city (113°56′59.08″ E, 22°31′18.51″ N), from intertidal sediment of a gravel beach, May 2014, X.M. Bian and W. Li (HMAS352505, holotype designated here, dried culture on SNA; culture ex-type CGMCC3.25346 = WL02375).

Sexual morph is not observed. Asexual morph on PDA and SNA conidiophores are solitary, often reduced to a single conidiogenous cell arising laterally from undifferentiated hypha, or stalked, bearing two or three conidiogenous cells at the top. Conidiogenous cells are annellidic, lateral or terminal, hyaline, smooth and thin walled, cylindrical to flask-shaped, and 8–30 × 1.5–2.5 µm, with several distinct annellations at the top with age. Conidia aseptate, solitary, arranged in slimy masses, hyaline, ellipsoidal, flat at the base, smooth to slightly rough, and 7–16 × 3.5–7 µm.

Culture characteristics—colonies on PDA are 40–55 mm diam in 7 d at 25 °C, flat, felty to floccose, and pale olive green near the centre, with a white regular margin; the reverse is pale olive green near the centre and white near the margin. Colonies on OA are 50–65 mm diam in 7 d at 25 °C, flat and pale olive green near the centre, with a white regular margin; the reverse is pale olive green near the centre with white near the margin. Colonies on SNA are 30–40 mm diam in 7 d at 25 °C, flat, with scarce aerial mycelia and are pale olive green near the centre, with a white regular margin; the reverse is pale olive green near the centre, with a white regular margin.

Other examined isolates: CHINA, Guangdong Province, Shenzhen city (113°56′59.08″ E, 22°31′18.51″ N), from intertidal sediment of a gravels beach, May 2014, X.M. Bian and W. Li (WL06356); ibid. (WL06357).

**Figure 8:**
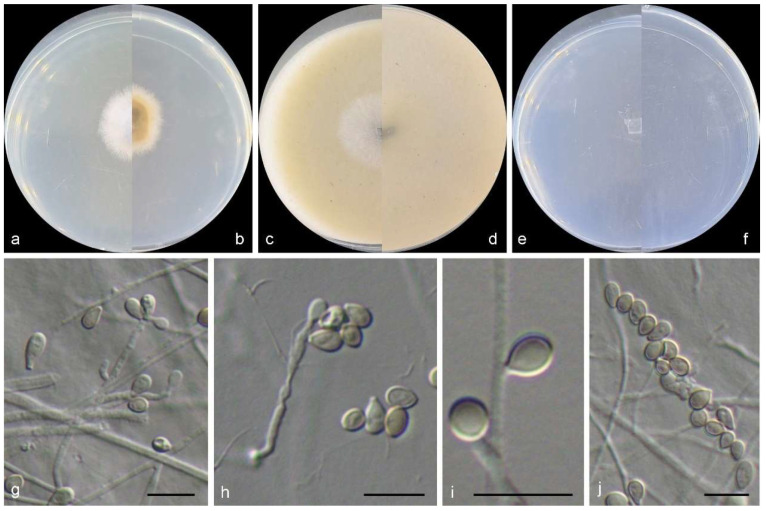
Morphological characters of *Scedosporium sphaerospermum* (from ex-type WL02796). (**a**–**f**): colonies on PDA, OA, and SNA after 7d; (**g**–**i**): Conidiophores and conidiogenous cells; (**j**): Conidia. Bars: (**g**–**j**) = 10 μm.

***Scedosporium sphaerospermum*** M.M. Wang, W. Li and L. Cai sp. nov., Figure 8.

FungalNames: FN 571627.

Etymology: referring to the globose shape of conidia.

Typus: CHINA, Shandong Province, Qingdao city (120°19′09.12″ E, 36°03′38.64″ N), from intertidal sediment of a mudflat, June 2014, X.M. Bian and W. Li (HMAS352506, holotype designated here, dried culture on SNA; culture ex-type CGMCC3.25347 = WL02796).

Sexual morph is not observed. Asexual morph on PDA and SNA conidiophores are solitary, sometimes reduced to a single conidiogenous cell arising laterally from undifferentiated hyphae. Conidiogenous cells are annellidic, lateral or terminal, hyaline, smooth- and thin walled, cylindrical to flask-shaped, and 2–20 × 1.5–2.5 µm, with several, distinct annellations at the top with the age. Conidia aseptate, solitary, arranged in slimy masses, hyaline to pale brown, globose to ellipsoidal, sometimes elliptic to oblong obovate, flat at the base, and 6–8 × 4–5 µm.

Culture characteristics—colonies on PDA are 15–25 mm diam in 7 d at 25 °C, flat, felty to floccose, and pale grey near the centre, with a white regular margin; the reverse is yellowish brown near the centre and white near the margin. Colonies on OA are 20–35 mm diam in 7 d at 25 °C, flat, and pale grey near the centre, with a white regular margin; the reverse is a pale greyish yellow near the centre and white near the margin. Colonies on SNA are 10–20 mm diam in 7 d at 25 °C, flat, with scarce aerial mycelia, and white; the reverse is white.

Other examined isolates: CHINA, Shandong Province, Qingdao city (120°19′09.12″ E, 36°03′38.64″ N), from intertidal sediment of a mudflat, June 2014, X.M. Bian and W. Li (WL06360); ibid. (WL06361).

Notes: phylogenetically, *S. sphaerospermum* forms a sister clade to *S. angustum* and *S. fusoideum* (Arx) McGinnis, A.A. Padhye and Ajello in the genus *Scedosporium* (Figure 2), and differs from the latter species by 36 bp and 129 bp in the LSU-ITS-*tef1*-*tub2* dataset, respectively. Morphologically, the three species could be distinguished by the characters of the sexual morph (absence in *S. sphaerospermum*, ascomata spherical, nonostiolate, dark brown, 100–150 µm, with allipsoidal or nearly cylindrical with rounded ends ascospores in *S. angustum* and ascomata submerged or semi-immersed, spherical or nearly so, non-ostiolate, dark brown, 50–100 µm, with broadly fusiform, and yellowish or straw-coloured ascospores in *S. fusoideum*), and shape and size of conidia (globose to ellipsoidal, 6–8 × 4–5 µm *S. sphaerospermum* vs. clavate or nearly cylindrical, 5–10 × 3–4.5 µm in *S. angustum* and clavate, 6–10 × 3.5–5 µm in *S. fusoideum*) [49].

***Scopulariopsis*** Bainier, Bull. Soc. mycol. Fr. 23(2): 98 (1907).

***Scopulariopsis brevicaulis*** (Sacc.) Bainier [as ‘brevicaule’], Bull. Soc. mycol. Fr. 23(2): 99 (1907).

Examined isolates: CHINA, Shandong Province, Qingdao city, from *Ulva pertusa* from a sand beach, October 2011, Y.T. Peng, C.H. Feng, and C.L. Li (WL00657); Fujian Province, Ningde city (120°03′39.98″ E, 26°52′51.89″ N), from intertidal sediment of a mudflat, June 2014, X.M. Bian and W. Li (WL03882).

***Wardomyces*** F.T. Brooks and Hansf., Trans. Br. Mycol. Soc. 8(3): 137 (1923).

***Wardomyces inflatus*** (Marchal) Hennebert, Trans. Br. Mycol. Soc. 51(5): 755 (1968).

Examined isolates: CHINA, Shandong Province, Weihai city, from intertidal sediment of a sand beach, June 2010, K.M. Sun and W. Li (WL00510); Liaoning Province, Huludao city (120°47′33.34″ E, 40°36′14.56″ N), from intertidal sediment of a sand beach, May 2014, X.M. Bian and W. Li (WL02318).

## 4. Discussion

A systemic survey of fungal resources in Chinese intertidal areas has been implemented by us since 2007, and more than 3000 fungal strains were successfully isolated from thousands of marine algae and sediment samples. Based on morphological and phylogenetic analyses, approximately 600 species have been identified from these fungal strains (unpublished data). Using brine shrimp, agricultural pests, and plant pathogens as targets, we have screened the bioactivities of 181 fungal strains and found more than half of them displayed marked insecticidal and antifungal activities [19]. These results are consistent with the notion that the intertidal zone is an ideal shelter for hosting numerous fungi that can produce diverse metabolic activities [19,50].

Most of the microascaceous fungi have been previously reported as saprobes in seawater and sediments, and on mangrove plants and marine algae [8,9,10]. They can also act as endophytes on marine algae and seaweeds or be isolated from the inner tissue of marine animals such as marine sponges [7,8,9,51] (Table 2). In this study, 18 microascaceous species (48 isolates) were retrieved from Chinese intertidal areas. According to the catalogue shown in Table 2, the number of marine microascaceous species increased from 22 to 33 under our efforts. The newly increased species include six new species (*Gamisa sedimenticola*, *Microascus algicola*, *M. gennadii*, *Scedosporium ellipsosporium*, *S. shenzhenensis,* and *S. sphaerospermum*) and five new records for the marine environment (*M. murinus*, *M. restrictus*, *M. verrucosus*, *S. hainanense*, and *Wardomyces inflatus*).

Although only a small proportion of microascaceous fungi were recovered (~3% of the total fungal species recovered), we observed a relatively wide distribution of these fungi across different geographic regions. This distribution ranges from the higher-latitude regions, such as the Liaoning and Hebei provinces, to the lower regions, such as Fujian and Guangdong provinces. Furthermore, diverse substrates were also found to host microascaceous fungi in this study, such as several marine algae and sediments from mudflats, sandy beaches, and gravel beaches. These findings are consistent with our previous investigation using a molecular approach [14] and research based on culturable methods [8,52,53,54,55], This also suggests that microascaceous fungi probably possess a wide geographical distribution, diverse habitats/hosts, and multiple ecological functions in the ocean.

Altogether, our results broaden the information related to the diversity, distribution, and habitat of microascaceous fungi in the marine environment. However, the potential ecological roles and bioactivities of these microascaceous fungi are currently unknown and need further investigation.

**Table 2:** Records of *Microascaceae* in the marine environment.

Species	Country/Region/Location	Known Habitats/Hosts	Recorded Database/Reference
*Acaulium acremonium*	na	na	MF
*Cephalotrichum microsporum*	China; Europe	unidentified brown algae, green algae, and red algae	WoRMS; this study
*Ce. nanum*	China; Europe	Intertidal sediment	WoRMS; this study
*Ce. purpureofuscum*	China	Marine algae *Sargassum canfusum*	This study
*Ce. stemonitis*	Europe	na	MF and WoRMS
*Gamsia sedimenticola* *	China	Intertidal sediment	This study
*Microascus algicola* *	China	Unidentified brown algae; marine algae *Ahnfeltiopsis flabelliformis*, *Colpomenia sinuosa*, *Grateloupia filicina*	This study
*M. croci*#	China; Hawaiian, Line, and Phoenix Islands	Marine algae *Blidingia minima*; coastal sands	[52]; This study
*M. gennadii* *	China	Intertidal sediment	This study
*M. murinus* +#	China	Intertidal sediment	This study
*M. paisii*	na	na	MF
*M. restrictus* +#	China	Intertidal sediment	This study
*M. senegalensis*	Senegal	Mangrove soil	[53]
*M. trigonosporus*	China, Europe; Hawaiian, Line, and Phoenix Islands	Marine algae *Cladophora* sp., *Ulva linza*, *Gracilaria textorii*; intertidal sediment; coastal sand	MF and WoRMS; [52]
*M. verrucosus* +#	China	Intertidal sediment	This study
*Petriella sordida*	na	na	MF
*Scedosporium boydii*	China	Intertidal sediment	MF; this study
*S. dehoogii*	China	Coastal sediment	[8]
*S. ellipsosporium* *	China	Intertidal sediment	This study
*S. hainanense* +	China	Intertidal sediment	This study
*S. marinum*	India	Decaying woody stem of *Suaeda monoica*	[54]
*S. shenzhenensis* *	China	Intertidal sediment	This study
*S. sphaerospermum* *	China	Intertidal sediment	This study
*Scopulariopsis brevicaulis*	China, Europe, Croatia; Hawaiian, Line, and Phoenix Islands	Marine sponge *Tethya aurantium*; marine algae *Ulva pertusa*; intertidal sediment; coastal sands	MF and WoRMS; [52]; This study
*Sc. Brumptii*	Hawaiian, Line, and Phoenix Islands	Coastal sands	MF; [52]
*Sc. Candida*	Egypt	Salt marshes	MF; [55]
*Sc. Coprophila*	Hawaiian, Line, and Phoenix Islands	Coastal seawater and sands	[52]
*Sc. Fusca*	Europe	na	WoRMS
*Sc. Halophilica*	na	na	MF and WoRMS
*Sc. Hibernica*	na	na	MF
*Wardomyces anomalus*	na	na	MF
*W. inflatus* *+*	China	Intertidal sediment	This study
*Yunnania carbonaria*	Hawaiian	Coastal seawater	[52]

Notes: new species described in this study are marked as “*”, new records for the marine environment with “+”, and new records for China with “#”. MS = the Marine Fungi database (https://www.marinefungi.org/ (accessed on 30 October 2023)), WoRMS = the World Register of Marine Species database (https://www.marinespecies.org/ (accessed on 30 October 2023)), na = no data available.

## Figures and Tables

**Table 1:** Strains examined in this study, with information on the source, origin and GenBank accessions of the sequences.

Species	Isolate and Status	Source	Origin	GenBank Accession Number			
				LSU	ITS	TEF	TUB
*Acaulium acremonium*	MUCL 8274 = CBS 104.65 ^ET^	Wheatfield soil	Schleswig-Holstein, Germany	KY852479	KY852468	--	--
*A. album*	CBS 539.85 ^ET^	Hair in dung in pole cat	The Netherlands	KY852484	KY852473	--	--
*Canariomyces arenarius*	CBS 507.74 ^IT^	Desert soil	Egypt	KM655383	KM655344	KM655474	MK926898
*Ca. microsporus*	CBS 276.74 ^IT^	Desert soil	Egypt	MH872590	MH860852	MN078437	MK926899
*Ca. notabilis*	CBS 548.83 ^T^	Litter of *Phoenix canariensis*	Egypt	MH873362	MH861648	MN078432	MK926902
*Ca. subthermophilus*	CBS 509.74 ^T^	Desert soil	Egypt	MH872612	MH860876	MN078436	MK926904
*Ca. vonarxii*	CBS 160.80 = NHL 2831 ^T^	Dried flower of Hibiscus	Sudan	--	--	MN078435	MK926905
*Cephalotrichum asperulum*	CBS 582.71 ^T^	Soil	Buenos Aires, Argentina	LN851007	LN850960	LN851061	LN851114
*Ce. brevistipitatum*	CBS 157.57 ^T^	Tuber	Wageningen, The Netherlands	LN851031	LN850984	LN851084	LN851138
*Ce. columnare*	CBS 159.66 ^T^	Dung of hare	Johannesburg, South Africa	LN851010	LN850963	LN851064	LN851117
*Ce. cylindricum*	UAMH 1348 ^T^	Seed of sorghum	Kansas, USA	LN851012	LN850965	LN851066	LN851119
*Ce. dendrocephalum*	CBS 528.85 ^T^	Cultivated soil	Basrah, Iraq	LN851013	LN850966	LN851067	LN851120
*Ce. gorgonifer*	UAMH 3585	Mushroom compost	Alberta, Canada	LN851025	LN850978	LN851078	LN851132
*Ce. guizhouense*	CGMCC 3.18330 ^T^	Air from cave	Guizhou, China	MF419758	MF419788	MF419728	MF434549
*Ce. hinnuleum*	CBS 289.66 ^T^	Dung of deer	Tasmania, Australia	LN851032	LN850985	LN851085	LN851139
*Ce. laeve*	CGMCC 3.18329 ^T^	Limestone from cave	Guizhou, China	MF419778	MF419808	MF419748	MF434569
*Ce. microsporum*	CBS 523.63 ^ET^	Wheatfield soil	Schleswig-Holstein, Germany	LN851014	LN850967	LN851068	LN851121
	WL00192	Unidentified red algae	Qingdao, Shandong, China	**OR339938**	**OR339892**	**OR347703**	**OR338658**
	WL00303	Unidentified green algae	Weihai, Shandong, China	**OR339939**	**OR339893**	**OR347704**	**OR338659**
	WL00305	Unidentified green algae	Weihai, Shandong, China	**OR339940**	**OR339894**	**OR347705**	**OR338660**
	WL00350	Unidentified brown algae	Weihai, Shandong, China	**OR339941**	**OR339895**	**OR347706**	**OR338661**
*Ce. nanum*	CBS 191.61 ^ET^	Dung of deer	Surrey, England	LN851016	LN850969	LN851070	LN851123
	WL03115	Intertidal sediment	Ningbo, Zhejiang, China	**OR339945**	**OR339899**	**OR347710**	**OR338662**
	WL03362	Intertidal sediment	Ningbo, Zhejiang, China	**OR339946**	**--**	**OR347711**	**--**
*Ce. oligotriphicum*	CGMCC 3.18328 ^T^	Limestone from cave	Guizhou, China	MF419771	MF419801	MF419741	MF434562
*Ce. purpureofuscum*	UAMH 9209	Indoor air	British Columbia, Canada	LN851018	LN850971	LN851072	LN851125
	WL06350	*Sargassum canfusum*	Shenzhen, Guangdong, China	**OR339942**	**OR339896**	**OR347707**	**OR338664**
	WL06351	*Sargassum canfusum*	Shenzhen, Guangdong, China	**OR339943**	**OR339897**	**OR347708**	**OR338665**
	WL02022	*Sargassum canfusum*	Shenzhen, Guangdong, China	**OR339950**	**OR339898**	**OR347709**	**OR338663**
*Ce. stemonitis*	CBS 103.19 ^NT^	Seed	Wageningen, Netherlands	LN850952	LN850951	LN850953	LN850954
*Ce. verrucisporum*	CBS 187.78	Sand dune soil	Katijk, The Netherlands	LN851033	LN850986	LN851086	LN851140
*Gamsia aggregata*	CBS 251.69 ^ET^	Dung of carnivore	USA	LM652500	LM652378	--	--
*G. columbina*	CBS 233.66 ^ET^	Sandy soil	Giessen, Germany	LN851039	LN850990	LN851092	LN851146
*G. dimera*	CBS 235.66 = ATCC 18887 = IMI 117371 = MUCL 6388 ^T^	Wheatfield soil	Schleswig-Holstein, Germany	--	MH858785	--	--
*G. kooimaniorum*	CBS 143185 ^T^	Garden soil	The Netherlands	--	NR_159824	--	--
*G. sedimenticola*	WL02722 = CGMCC3.25342 ^T^	Intertidal sediment	Qingdao, Shandong, China	**OR339947**	**OR339900**	**OR347712**	**OR338666**
	WL06358	Intertidal sediment	Qingdao, Shandong, China	**OR339948**	**OR339901**	**OR347713**	**OR338667**
	WL06359	Intertidal sediment	Qingdao, Shandong, China	**OR339949**	**OR339902**	**OR347714**	**OR338668**
*G. simplex*	CBS 546.69 ^IT^	Milled Oryza sativa	Japan	LM652501	LM652379	LN851094	LN851148
*Graphium penicillioides*	CBS 102632 ^T^	*Populus nigra*	Czech Republic	KY852485	KY852474	--	--
*Kernia columnaris*	CBS 159.66 = IMI 116691 ^T^	Dung of hare	South Africa	KY852486	KY852475	KY852478	KY852477
*K. nitida*	CBS 282.52 = NBRC 8200	*Chrysolina sanguinolenta*	France	KY852487	KY852476	--	--
*Microascus algicola*	WL00347	Unidentified brown algae	Weihai, Shandong, China	**OR339954**	**OR339907**	**OR347719**	**OR338669**
	WL00633	*Colpomenia sinuosa*	Qingdao, Shandong, China	**OR339955**	**OR339908**	**OR347720**	**OR338670**
	WL01268 = CGMCC3.25343 ^T^	*Grateloupia filicina*	Qingdao, Shandong, China	**OR339962**	**OR339909**	**OR347721**	**OR338672**
	WL01021	*Ahnfeltiopsis flabelliformis*	Qingdao, Shandong, China	**OR339957**	**OR339910**	**OR347722**	**OR338671**
*M. campaniformis*	CBS 138126 = UTHSC 10-565 = FMR 12343 ^T^	Human BAL	USA	HG380495	LM652391	HG380418	LM652606
*M. cinereus*	FMR 12217 = UTHSC 10-2805 ^NT^	Human BAL	USA	--	LM652397	--	LM652611
*M. croci*	CBS 158.44 = MUCL 9002 ^T^	Crocus sp.	Lisse, The Netherlands	LM652508	LM652407	LM652560	LM652621
	WL02042	*Blidingia minima*	Qingdao, Shandong, China	**OR339982**	**OR339903**	**OR347715**	**OR338673**
*M. expansus*	CBS 138127 = UTHSC 06-4472 = FMR 12266 ^T^	Human sputum	USA	HG380492	LM652410	HG380415	LM652624
*M. gennadii*	WL02353 = CGMCC3.25344 ^T^	Intertidal sediment	Unknown	**OR339971**	**OR339904**	**OR347716**	**OR338674**
	WL06354	Intertidal sediment	Unknown	**OR339952**	**OR339905**	**OR347717**	**OR338675**
	WL06355	Intertidal sediment	Unknown	**OR339953**	**OR339906**	**OR347718**	**OR338676**
*M. gracilis*	CBS 369.70 ^IT^	Food	Japan	HG380467	LM652412	HG380390	LM652625
*M. hyalinus*	CBS 766.70 ^IT^	Dung of cow	USA	LM652513	LM652418	LM652564	LM652631
*M. intricatus*	CBS 138128 = UTHSC 07-156 = FMR 12264 ^T^	Human BAL	USA	HG380496	LM652419	HG380419	LM652632
*M. longirostris*	CBS 196.61 = MUCL 9058 ^NT^	Wasp’s nest	Maine, USA	LM652515	LM652421	LM652566	LM652634
*M. murinus*	CBS 830.70 = IHEM 18567 ^T^	Composed municipal waste	Giessen, Germany	HG380481	LM652424	HG380404	LM652637
	WL05483	Intertidal sediment	Qingdao, Shandong, China	**OR339959**	**OR339912**	**OR347724**	**OR338678**
	WL03684	Intertidal sediment	Yantai, Shandong, China	**OR339958**	**OR339911**	**OR347723**	**OR338677**
*M. paisii*	UTHSC 07-639 = FMR 12263	Human BAL	USA	HG380451	LM652425	HG380374	LM652638
*M. pyramidus*	CBS 212.65 ^IT^	Desert soil	California, USA	HG380435	LM652439	HG380358	LM652652
*M. restrictus*	CBS 138277 = UTHSC 09-2704 = FMR 12227 ^T^	Human left hallux	USA	HG380494	LM652440	HG380417	LM652653
	WL02392	Intertidal sediment	Huludao, Liaoning, China	--	--	**OR347725**	**OR338679**
*M. senegalensis*	CBS 277.74 = IHEM 18561 ^T^	Mangrove soil	Senegal	LM652523	LM652441	LM652574	LM652654
*M. sparsimycelialis*	LC12478 ^T^	Animal faeces	Laibin, Guangxi, China	--	--	MK336046	MK336124
*M. superficialis*	LC12597 ^T^	Animal faeces	Laibin, Guangxi, China	--	--	MK336048	MK336126
*M. trigonosporus*	CBS 218.31 ^T^	na	USA	HG380436	LM652443	HG380359	LM652655
	WL00670	*Cladophora* sp.	Qingdao, Shandong, China	**OR339961**	**OR339916**	**OR347729**	**OR338683**
	WL01853	*Ulva linza*	Qingdao, Shandong, China	**OR339963**	**OR339917**	**OR347730**	**OR338684**
	WL01882	*Gracilaria textorii*	Qingdao, Shandong, China	**OR339944**	**OR339918**	**OR347731**	**OR338685**
	WL02833	Intertidal sediment	Qingdao, Shandong, China	**OR339964**	**OR339919**	**OR347732**	**OR338686**
*M. trigonus*	LC12520 ^T^	Soil	Guilin, Guangxi, China	--	--	MK336052	MK336130
*M. verrucosus*	CBS 138278 = UTHSC 10-2601 = FMR 12219 ^T^	Human, BAL fluid	USA	HG380493	LM652446	HG380416	LM652658
	WL02828	Intertidal sediment	Dongying, Shandong, China	**OR339965**	**OR339920**	**OR347733**	**OR338687**
*Microascus* sp.	WL01046	*Grateloupia filicina*	Qingdao, Shandong, China	**OR339956**	**OR339913**	**OR347726**	**OR338680**
	WL06352	*Grateloupia filicina*	Qingdao, Shandong, China	**--**	**OR339914**	**OR347727**	**OR338681**
	WL06353	*Grateloupia filicina*	Qingdao, Shandong, China	**--**	**OR339915**	**OR347728**	**OR338682**
*Parascedosporium tectonae*	CBS 127.84 ^T^	Human BAL	USA	EF151332	--	--	--
*Petriella sordida*	CBS 124169	Corner of a bathroom	The Netherlands	AY281099	GQ426957	--	--
*Pithoascus ater*	CBS 400.34 = IHEM 18608 ^T^	na	na	LM652526	LM652447	LM652576	LM652659
*Pi. exsertus*	CBS 819.70 ^T^	*Megachile willoughbiella*	Tastrup, Denmark	LM652528	LM652449	LM652578	LM652661
*Pi. intermedius*	CBS 217.32 ^T^	Root of *Fragaria vesca*	North Carolina, USA	LM652529	LM652450	LM652579	LM652662
*Pi. nidicola*	CBS 197.61 ^ET^	*Dipodomys merriami*	Utah, USA	LM652530	LM652451	LM652580	LM652663
*Pi. stoveri*	CBS 176.71 ^T^	Root of Beta vulgaris	Ohio, USA	LM652532	LM652453	LM652581	LM652664
*Pseudoscopulariopsis asperispora*	LC12445 ^T^	Animal faeces	Guilin, Guangxi, China	--	--	MK336064	MK336142
*Ps. hibernica*	UAMH 2643 = ATCC 16690	Soil	Ireland	LM652533	LM652454	LM652582	LM652665
*Ps. schumacheri*	CBS 435.86 ^NT^	Soil	Puerto de la Quesera, Spain	LM652534	LM652455	LM652583	LM652666
*Rhinocladium lesnei*	CBS 108.10	Foot of human	France	MH866122	MH854594	--	--
*Scedosporium angustum*	CBS 254.72 ^ET^	Sewage half digestion tank	Ohio, USA	--	AJ888414	--	AJ889604
*S. apiospermum*	FMR 8619 ^ET^	Keratitis of huma	Brazil	--	NR_130664	--	AJ889584
*S. aurantiacum*	IMI 392886 ^T^	Ulcer on ankle of human	Spain	--	AJ888440	--	AJ889597
*S. boydii*	CBS 101.22 = IMI 015407 = JCM 7441 = NCPF 2216 = UAMH 3982 ^T^	Mycetoma	USA	EF151320	KT008518	KT069589	KT008455
	WL02794	Intertidal sediment	Qingdao, Shandong, China	**OR339968**	**OR339923**	**OR347736**	**OR338688**
	WL05602	Intertidal sediment	Yantai, Shandong, China	**OR339969**	**OR339924**	**OR347737**	**OR338689**
	WL05951	Intertidal sediment	Tianjin, China	**OR339970**	**OR339925**	**OR347738**	**OR338690**
*S. cereisporum*	FMR 12996 ^ET^	Wastewater sludge	Mûrs-Erignés, France	--	KJ599660	--	KJ599659
*S. dehoogii*	CBS 117406 ^ET^	Garden soil	Barcelona, Spain	--	KT163400	--	KT163401
*S. desertorum*	CBS 489.72 ^ET^	Salt-marsh soil	Kuwait	--	--	--	AM409106
*S. ellipsoideum*	CBS 418.73 ^ET^	Soil	Tayikistán	--	AJ888426	--	AJ889595
*S. ellipsosporium*	WL02370 = CGMCC3.25345 ^T^	Intertidal sediment	Shenzhen, Guangdong, China	**OR339973**	**OR339926**	**OR347739**	**OR338692**
	WL02793	Intertidal sediment	Qingdao, Shandong, China	**OR339972**	**OR339927**	**OR347740**	**OR338693**
*S. fusoideum*	CBS 106.53 ^ET^	Goat dung Aligarh	India	--	AJ888428	--	AJ889601
*S. haikouense*	CGMCC 3.20468 = GZUIFR 21.833 ^T^	Green-belt soil	Haikou, Hainan, China	--	MZ469289	--	MZ488563
*S. hainanense*	CGMCC 3.20469 = GZUIFR 21.829 ^T^	Green-belt soil	Sanya, Hainan, China	--	MZ469285	--	MZ488559
	WL05931	Intertidal sediment	Yancheng, Jiangsu, China	**OR339967**	**OR339922**	**OR347735**	**OR338691**
*S. minutisporum*	FMR 4072 ^ET^	River sediment Tordera river	Spain	--	AJ888384	--	AJ889592
*S. multisporum*	CGMCC 3.20470 = GZUIFR 21.830 ^T^	Green-belt soil	Huaihua, Hunan, China	--	MZ469286	--	MZ488560
*S. rarisporum*	GZUIFR-G79 ^T^	Root soil of *Wodyetia bifurcata*	Guigang, Guangxi, China	--	KX790702	--	--
*S. shenzhenensis*	WL02375 = CGMCC3.25346 ^T^	Intertidal sediment	Shenzhen, Guangdong, China	**OR339960**	**OR339928**	**OR347741**	**OR338694**
	WL06356	Intertidal sediment	Shenzhen, Guangdong, China	**OR339974**	**OR339929**	**OR347742**	**OR338695**
	WL06357	Intertidal sediment	Shenzhen, Guangdong, China	**OR339975**	**OR339930**	**OR347743**	**OR338696**
*S. sphaerospermum*	WL02796 = CGMCC3.25347 ^T^	Intertidal sediment	Qingdao, Shandong, China	**OR339976**	**OR339931**	**OR347744**	**OR338698**
	WL06360	Intertidal sediment	Qingdao, Shandong, China	**OR339977**	**OR339932**	**OR347745**	**OR338699**
	WL06361	Intertidal sediment	Qingdao, Shandong, China	**OR339978**	**OR339933**	**OR347746**	**OR338700**
*Scedosporium* sp.	WL02426	Intertidal sediment	na	**OR339966**	**OR339921**	**OR347734**	**OR338697**
*Scopulariopsis africana*	CBS 118736 ^T^	Mud, salt pan	South Africa	KX924040	KX923954	KX924176	KX924388
*Sc. albida*	CBS 119.43 ^T^	Soil	The Netherlands	LN850849	LN850800	LM652592	LN850897
*Sc. alboflavescens*	CBS 399.34 ^T^	Diseased skin	Austria	LM652539	KX923956	KX924179	JQ434537
*Sc. asperula*	CBS 853.68	Compost soil	Germany	JQ434669	LM652461	JQ434621	JQ434558
*Sc. brevicaulis*	MUCL 40726 ^T^	Indoor air	Alberta, Canada	HG380440	LM652465	HG380363	LM652672
	WL00657	*Ulva pertusa*	Qingdao, Shandong, China	**OR339979**	**OR339934**	**OR347747**	**OR338701**
	WL03882	Intertidal sediment	Ningde, Fujian, China	**OR339980**	**OR339935**	**OR347748**	**OR338702**
*Sc. candida*	MUCL 40743 ^ET^	Indoor air	Canada	HG380458	LM652484	HG380381	LM652690
*Sc. caseicola*	CBS 480.62 ^T^	Cheese coating	The Netherlands	KX924041	KX924020	KX924247	KX924454
*Sc. cordiae*	FMR 12338 ^T^	Human finger	USA	HG380499	LM652491	HG380422	LM652673
*Sc. flava*	CBS 207.61 = MUCL 9031 ^NT^	Cheese	UK	HG380464	LM652493	HG380387	LM652697
*Sc. macurae*	CBS 506.66 ^T^	Chicken litter	Canada	LN850854	LN850805	KX924250	LN850902
*Sc. sexualis*	CBS 250.64 ^T^	*Oryza sativa*	Burma	KX924042	KX924024	KX924251	KX924458
*Sc. soppii*	UAMH 9169 ^T^	Wood of *Populus tremuloides*	Alberta, Canada	LM652552	LM652495	LM652595	LM652698
*Wardomyces anomalus*	CBS 299.61 ^ET^	Air cell of egg	Ontario, Canada	MH869626	MH858058	LN851095	LN851149
*W. giganteus*	CBS 746.69 ^T^	Insect frass in dead log	Ontario, Canada	MH871180	MH859408	LN851096	LN851150
*W. inflatus*	CBS 216.61 ^IT^	Wood, *Acer* sp.	Quebec, Canada	LM652553	LM652496	LN851098	--
	WL02318	Intertidal sediment	Huludao, Liaoning, China	**OR339951**	**OR339937**	**OR347750**	**OR338704**
	WL00510	Intertidal sediment	Weihai, Shandong, China	**OR339981**	**OR339936**	**OR347749**	**OR338703**
*W. ovalis*	CBS 234.66 = IMI 117372 = MUCL 6031 ^T^	Wheatfield soil	Schleswig-Holstein, Germany	LN851050	MH858784	LN851101	LN851155
*W. pulvinatus*	CBS 112.65 ^IT^	Salt marsh	Cheshire, England	MH870142	MH858508	LN851102	LN851156
*Wardomycopsis dolichi*	LC12503 ^T^	Soil	Guilin, Guangxi, China	MK329043	MK329138	MK336073	--
*Wa. fusca*	LC12476 ^T^	Soil	Guilin, Guangxi, China	MK329047	MK329142	MK336077	MK336148
*Wa. humicola*	CBS 487.66 ^T^	Soil	Ontario, Canada	LM652554	LM652497	--	--
*Wa. inopinata*	FMR 10305	Soil	Myanmar	LM652555	LM652498	--	--
*Wa. litoralis*	CBS 119740 ^T^	Beach soil	Castellon, Spain	LN851055	LN851000	LN851107	LN851161
*Wa. longicatenata*	CGMCC 3.17947 ^T^	Air	Guizhou, China	KU746756	KU746710	KX855255	KU746801
*Yunnania carbonaria*	CBS 205.61 ^T^	Soil	Panama	HG380462	KX923820	KX924044	KX924254
*Y. penicillata*	CBS 130296 ^T^	Moulded pork sample	China	KY659809	JN831361	KY659808	KY659807
*Y. smithii*	CBS 855.68 ^T^	Garden soil	Germany	KX924028	KX923822	KX924046	KX924256

Notes: ^T^ = ex-type, ^ET^ = epi-type, ^IT^ = iso-type, ^NT^ = neo-type. Sequences newly generated in this study are marked in bold.

## Data Availability

All sequences generated in this study were submitted to GenBank (https://www.ncbi.nlm.nih.gov, accessed on 25 December 2023).
